# FDG-PET/CT in colorectal cancer: potential for vascular-metabolic imaging to provide markers of prognosis

**DOI:** 10.1007/s00259-021-05318-y

**Published:** 2021-04-10

**Authors:** Shih-hsin Chen, Kenneth Miles, Stuart A. Taylor, Balaji Ganeshan, Manuel Rodriquez, Francesco Fraioli, Simon Wan, Asim Afaq, Robert Shortman, Darren Walls, Luke Hoy, Raymond Endozo, Aman Bhargava, Matthew Hanson, Joseph Huang, Sherif Raouf, Daren Francis, Shahab Siddiqi, Tan Arulampalam, Bruce Sizer, Michael Machesney, Nicholas Reay-Jones, Sanjay Dindyal, Tony Ng, Ashley M Groves

**Affiliations:** 1 Division of Medicine, Research Department of Imaging, University College London (UCL), London, UK; 2 Department of Nuclear Medicine, Keelung Chang Gung Memorial Hospital, Keelung, Taiwan; 3 Centre for Medical Imaging, University College London, London, UK; 4 University College London Hospitals (UCLH) NHS Foundation Trust, Surgery and Cancer Board, Imaging Division, University College Hospital (UCH), London, UK; 5 Department of Research Pathology, Cancer Institute, UCL, London, UK; 6 University of Iowa, Carver College of Medicine, Iowa City, USA; 7 Institute of Health Barts and London Medical School, Queen Mary University of London (QMUL), London, UK; 8 Barking, Havering and Redbridge University Hospitals NHS Trust, Division of Cancer and Clinical Support, Queens and King George Hospitals, Essex, UK; 9 Radiotherapy Department, Barts Cancer Centre, St Bartholomew’s Hospital, West Smithfield, London, UK; 10 Royal Free London NHS Foundation Trust, Department of Colorectal Surgery, Barnet and Chase Farm Hospitals, London, UK; 11 Mid Essex Hospital Services NHS Trust, Department of Lower GI Surgery and Coloproctology, Broomfield Hospital, Essex, UK; 12 East Suffolk and North Essex NHS Foundation Trust, Department of Surgery & Department of Clinical Oncology, Colchester General Hospital, Essex, UK; 13 Barts Health NHS Trust, Cancer Clinical Board, Colorectal Surgery, Whipps Cross Hospital, London, UK; 14 East and North Hertfordshire NHS Trust, Colorectal Surgery, Queen Elizabeth II Hospital, Hertfordshire, UK; 15 East and North Hertfordshire NHS Trust, Colorectal Surgery, Lister Hospital, Hertfordshire, UK; 16 School of Cancer & Pharmaceutical Sciences, Kings College London (KCL), London, UK

**Keywords:** FDG-PET, CT perfusion, Colorectal cancer, Vascular, Metabolism, Survival

## Abstract

**Purpose:**

This study assesses the potential for vascular-metabolic imaging with FluoroDeoxyGlucose (FDG)–Positron Emission Tomography/Computed Tomography (PET/CT) perfusion to provide markers of prognosis specific to the site and stage of colorectal cancer.

**Methods:**

This prospective observational study comprised of participants with suspected colorectal cancer categorized as either (a) non-metastatic colon cancer (M0colon), (b) non-metastatic rectal cancer (M0rectum), or (c) metastatic colorectal cancer (M+). Combined FDG-PET/CT perfusion imaging was successfully performed in 286 participants (184 males, 102 females, age: 69.60 ± 10 years) deriving vascular and metabolic imaging parameters. Vascular and metabolic imaging parameters alone and in combination were investigated with respect to overall survival.

**Results:**

A vascular-metabolic signature that was significantly associated with poorer survival was identified for each patient group: M0colon – high Total Lesion Glycolysis (TLG) with increased Permeability Surface Area Product/Blood Flow (PS/BF), Hazard Ratio (HR) 3.472 (95% CI: 1.441–8.333), *p* = 0.006; M0rectum – high Metabolic Tumour Volume (MTV) with increased PS/BF, HR 4.567 (95% CI: 1.901–10.970), *p* = 0.001; M+ participants, high MTV with longer Time To Peak (TTP) enhancement, HR 2.421 (95% CI: 1.162–5.045), *p* = 0.018. In participants with stage 2 colon cancer as well as those with stage 3 rectal cancer, the vascular-metabolic signature could stratify the prognosis of these participants.

**Conclusion:**

Vascular and metabolic imaging using FDG-PET/CT can be used to synergise prognostic markers. The hazard ratios suggest that the technique may have clinical utility.

## Introduction

Colorectal cancer is the second commonest cause of cancer-related mortality in Western societies [1]. It is well established that the survival of patients with this disease is related to tumour stage [2]. However, even patients with the same tumour stage may differ in clinical course [3, 4]. Additional markers of prognosis therefore may enable further sub-stratification of patients to optimise treatment for individual sub-groups.

The biological characteristics and treatments for colorectal cancer vary according to tumour location (i.e. colon versus rectum) and tumour stage. Cancers of the rectum and colon exhibit differences in their gene expression profiles and carcinogenesis pathways [5]. The biological status of the tumour also changes as it progresses between stages [6]. This biological diversity is reflected by the use of distinct treatments for each situation. For colon cancer patients, the management principle is surgical resection when possible [7], while rectal cancer patients frequently receive neoadjuvant radiotherapy or chemoradiotherapy prior to surgery [8]. For patients with early disease, surgery offers the prospect of cure. Patients with more advanced disease may require neoadjuvant treatment, if the tumour is located in the rectum, or adjuvant chemotherapy if located in the colon [9]. Patients with metastatic disease may benefit from prolonged survival with systemic therapy, irrespective of the location of the primary tumour [9]. The potential therapeutic impact of prognostic markers will therefore be different for each of these situations. Thus, there is a need for prognostic markers specific to each clinical scenario.

Medical imaging characterizes human tissue non-invasively, providing information that frequently contributes to treatment decisions for patients with colorectal and other cancers [10]. In addition to providing information for cancer diagnosis and staging, incorporation of quantitative imaging techniques into routine clinical examinations can potentially provide complementary prognostic information [11]. A tumour microenvironment, such as immune cell infiltration, was shown to be a better predictor of patient prognosis than conventional histopathological results [12]. Quantitative medical imaging features might be able to foretell these differences, such as tumours with or without gene mutations. These are important to the disease management and any patient outcomes [13]. Moreover, imaging can provide this information at an earlier stage in the clinical pathway than tissue-based prognostic markers. These tissue markers require histological examination of surgical specimens, such as tumour grade and differentiation, the presence of extramural vascular invasion, tumour budding and the number of lymph nodes harvested [14]. Thus, imaging markers of prognosis obtained pre-operatively can potentially inform the selection of patients for neo-adjuvant treatment.

FluoroDeoxyGlucose (FDG)–Positron Emission Tomography/Computed Tomography (PET/CT) is an imaging technique that offers multi-parametric imaging for oncology patients [15]. In colorectal cancer, FDG-PET/CT is effective in the detection of metastatic disease [16]. In a series of 107 participants treated with surgery, those with lower tumour uptake of FDG, measured as the maximum Standardized Uptake Value (SUV_max_) demonstrated longer survival [17]. Another study consisting of 231 participants showed superior survival in those tumours which exhibited a lower Metabolic Tumour Volume (MTV) [18]. A meta-analysis found that participants with liver metastases from colorectal cancer and with a high SUV_max_ value, had poorer overall survival [19]. In addition, the CT component of PET/CT can provide prognostic information through the application of perfusion imaging to assess tumour vascular support [20]. Higher tumour Blood Flow (BF) was found in participants with disease-free status, compared to those with subsequent metastasis after surgery in a series with 52 colorectal participants [21]. Lower tumour BF was shown to be associated with poorest survival in a report comprising of 44 participants who were treated with surgery alone [22]; and with treatment failure in another study consisting of 24 participants receiving chemoradiation [23]. Combined vascular-metabolic parameters that assess the degree to which tumour vascularity and metabolism are coupled, can further enhance this multi-parametric approach to prognostic imaging [15].

This study assesses the potential for vascular-metabolic imaging with FDG-PET/CT to provide markers of prognosis specific to participants with (a) non-metastatic colon cancer (M0colon), (b) non-metastatic rectal cancer (M0rectum) and (c) metastatic colorectal cancer (M+).

## Materials and methods

### Study design and participant cohort

This prospective observational study was approved by the institution’s research ethics committee. Participants with suspected colorectal cancer on the basis of sigmoidoscopy, colonoscopy and/or CT images were recruited from several local hospitals, after giving informed consent. Based on clinical, pathological and imaging findings, participants were categorized as either (a) M0colon, (b) M0rectum, or (c) M+. Participants underwent combined FDG-PET and CT perfusion imaging. After imaging, the participants were treated as per the decisions made by their local multi-disciplinary team and followed-up at their referring hospitals.

In this study, overall survival is defined as, ‘the time between imaging and the time of death or last clinical follow-up was determined from review of clinical records’.

### Image acquisition and analysis

All the FDG-PET/CT studies were performed on a PET/64-detector-CT (Discovery VCT, General Electric (GE) Healthcare, Amersham, UK) at a single institution. The imaging system had undergone regular quality control and quality assurance, and was accredited for quantitative FDG imaging by the UK PET Core Lab [24]. Participants were requested to fast for at least 4 h prior to tracer injection and received an injected activity of (250 ± 80) Megabecquerels (MBq) of FDG. Image acquisition began at 66 ± 7 min after injection. Low-dose CT for attenuation correction was acquired with 64 × 3.75 mm detectors, a pitch of 1.5 and 5 mm collimation [140 k voltage peak (kVp), 40 mA per second (mAs), in 0.8 s]. PET was acquired in 2-dimensional (2D) mode, with 4 min/bed position from skull to upper thigh. Images were reconstructed using Ordered Subsets Expectation Maximization (OSEM) with two iterations and 28 subsets, with a slice thickness of 3.27 mm. The axial field of view was 100 cm. Perfusion CT was performed immediately after the PET acquisition. 50 mL of intravenous iodinated contrast was given at a rate of 5 mL/s (350 mg/mL iodine Omnipaque, GE Healthcare; Chalfont St Giles, UK), followed by 50 mL of normal saline at the same speed. After a delay of 10 s, CT images were acquired without table movement with acquisition parameters of 120 kVp, 60 mAs using 64 × 0.6 mm detectors (4-cm coverage), 2-s interval for 20 frames, then 5-s interval for 22 frames. The Display Field Of View (DFOV) was 36 cm. The total acquisition time for CT perfusion was 150 s. CT images were reconstructed using the standard reconstruction soft tissue kernel and a filtered-back projection algorithm.

The tracer uptake within the tumour was semiquantified using the mean and maximum standardized uptake value, calculated as tissue concentration in a Volume of Interest (VOI) (MBq)/kg/[injected dose (MBq)/body weight (kg)]. The VOI was determined by manually placing the centre seed point at the site of the most intense uptake and the using of a 40% SUV_max_ contour to grow the volume by the PET Volume Computer Assisted Reading (VCAR) program on the GE Advantage Workstation (GE Healthcare, Chalfront St Giles, UK). Manual adjustment was applied when necessary to avoid nearby high physiologic uptake such as the bladder. In addition, Total Lesion Glycolysis (TLG) and MTV were calculated by the program.

**Fig. 1:**
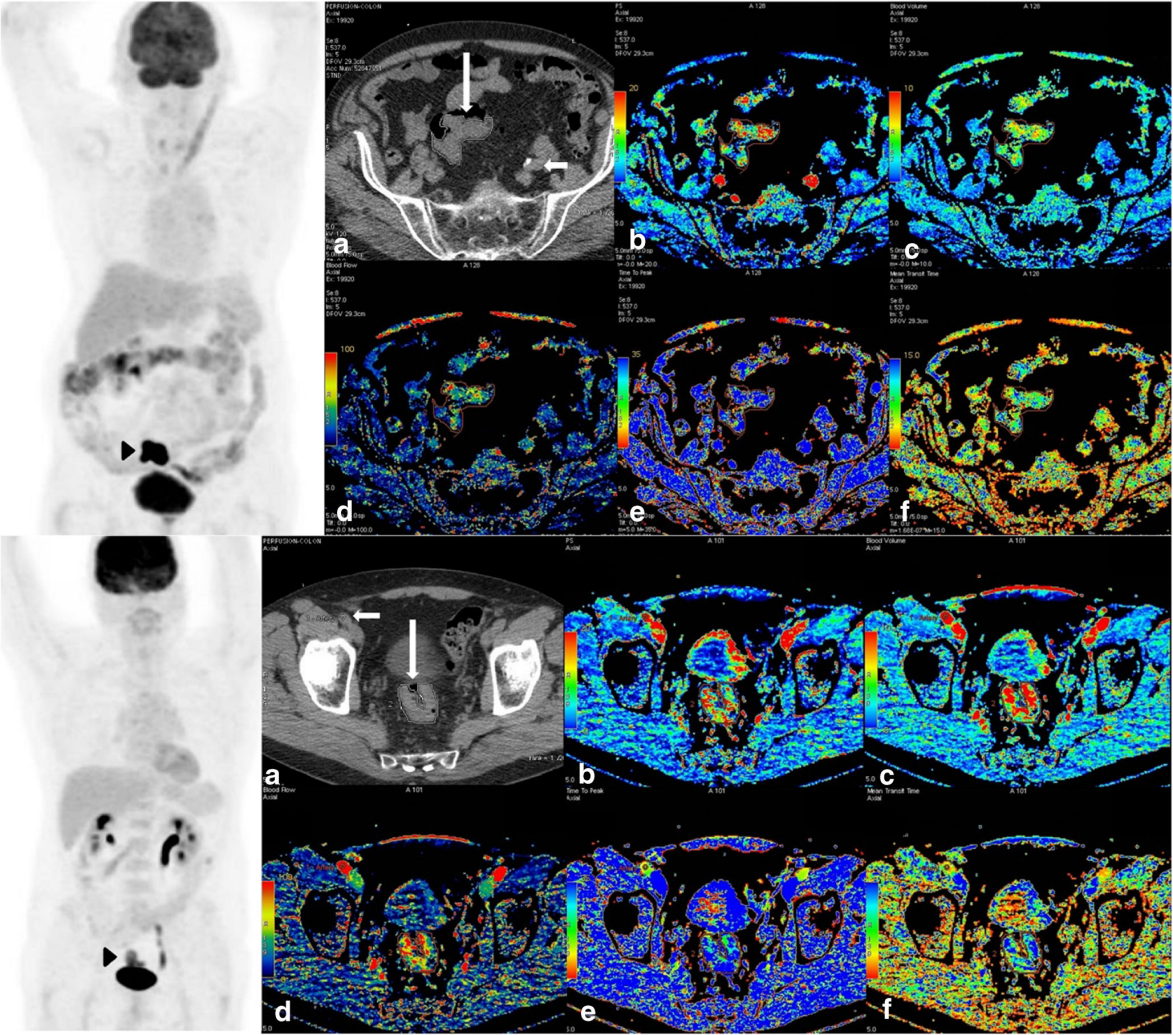
Illustrative coronal FDG-PET Maximum Intensity Projection (MIP), conventional CT and perfusion CT images from two patients with T3N0M0 sigmoid colon cancer; an 82-year-old who died 14.8 months after scan (upper row), and a 69-year-old who remained alive 40 months after scan (lower row). In each case, the position of the tumour was identified on the FDG-PET maximum intensity projection images (dark arrowheads). The positions of artery (arterial input, white short arrow) and tumour (white long arrow) were selected on conventional axial CT images (**a**). The software would then calculate the following CT perfusion parameters displayed as parametric maps: permeability surface area product (**b**), blood volume (**c**), blood flow (**d**), time to peak (**e**), and mean transit time (**f**). The average values of each pixel within the tumour were recorded. For the patient with poorer survival (upper row), the TLG (290.44) and PS/BF (0.26) were both above the median value (176.51 and 0.17). The TLG (64.02) and PS/BF (0.15) were both below the median for the patient who survived longer (TLG, Total Lesion Glycolysis, PS, Permeability Surface area product, BF, Blood Flow)

The perfusion CT data was analysed using a commercial software that implements a distributed parameter analysis (Perfusion 4D; GE Healthcare, Chalfont St Giles, UK). The processing threshold was set between 0 and 120 Hounsfield Units (HU). An arterial time-enhancement curve was derived by placing a circular region of interest within the best-visualized artery. A tumour region of interest was defined by depicting the contour of tumour in all images where the tumour was visible. No motion correction was applied. Parametric maps of tumour Blood Flow (BF), Blood Volume (BV), Mean Transit Time (MTT), Time To Peak (TTP) enhancement and Permeability Surface area product (PS) were generated and the mean values of these parameters recorded (Fig. 1). The ratio of PS/BF was calculated as an index of the extraction efficiency for contrast material within the tumour [20]. Both the PET and perfusion CT parameters were based on the primary tumour. The operator was blinded to the participant’s outcome.

### Inter-observer agreement

The inter-observer agreement of the image analysis for derivation of tumour vascular and metabolic parameters was evaluated. This was performed by comparing the measurements from 25 randomly selected participants. The analysis was performed by a nuclear medicine physician with 4 years of experience and by a nuclear medicine technologist with 20 years of experience. The agreement between paired measurements was expressed as the Intra-Class Correlation Coefficient (ICC) using a two-way random effect model [25], and also as the Coefficient of Variation (COV).

### Statistical analysis

Tumour vascular and metabolic parameters from M0colon, M0rectum and M+ participants were compared using the Student’s *t* test. The inter-relationships between vascular-metabolic imaging characteristics and clinical features were initially explored using unsupervised hierarchical cluster analysis with Euclidian distance and complete linkage (imaging parameters normalized using the z-score). A heat map was used to display the clustered imaging parameters with participants sorted by average z-score, along with the corresponding tumour stage, site (rectum versus colon) and survival greater or less than 60 months for each patient.

For each clinical group, image parameters from FDG-PET and CT perfusion were dichotomized by their median values. Kaplan–Meier analysis was used to estimate the overall survival of participants with tumour values above or below the median. Survival differences were assessed for statistical significance using the log-rank test. Cox regression was used to estimate the hazard ratio of the prognosticators. The vascular (CT perfusion) and metabolic (FDG-PET) parameters that best differentiates poor prognostic participants from good prognostic participants (the lowest *p* value even if not significant) for each of the three clinical sub-groups were chosen. The median values of both parameters (best metabolic and best vascular) were used to stratify participants within the respective clinical sub-group into four quadrants/sub-groups (low-low, low-high, high-low, high-high), and compared whether one sub-group had different prognosis as compared to other three categories.

Significant clinical parameters and the vascular metabolic profile were entered into the multivariate analysis. The possibility of effect-size bias resulting from the selection of prognostic thresholds for combined vascular-metabolic parameters was minimized using 5-fold cross validation [26]. A *p* value <0.05 was considered to be significant. All statistical analyses were run on IBM SPSS Statistics Version 22.0 (Armonk, NY:IBM Corps).

## Results

**Fig. 2:**
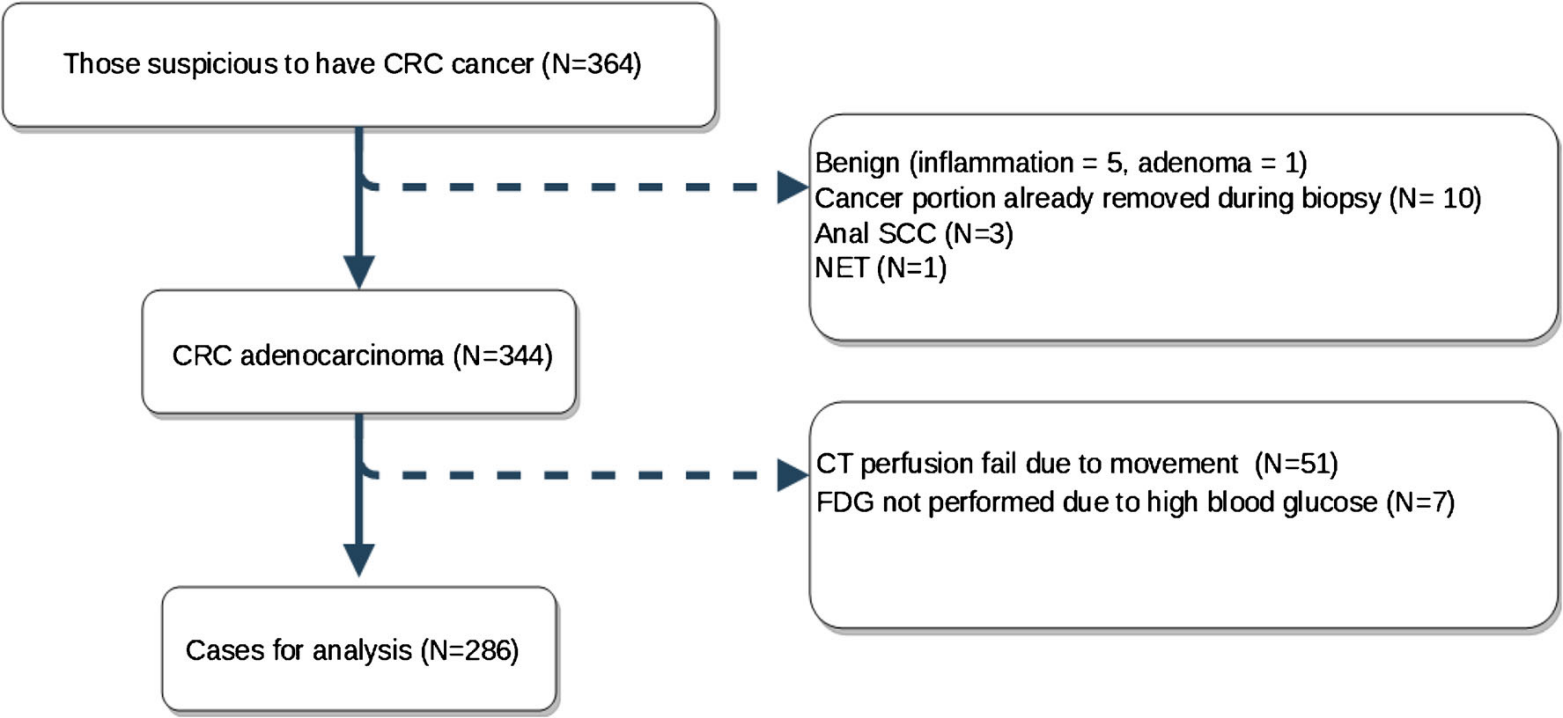
The flow diagram of patient recruitment. Amongst the 364 recruited participants, some were found to have benign histologies or other cancer types. Only 344 participants had colorectal adenocarcinoma visible on the imaging studies. CT perfusion could not be analysed in 51 participants because of motion artefacts. FDG-PET was not performed in 7 participants due to high blood sugar. (CRC, Colorectal Cancer; SCC, Squamous Cell Carcinoma; NET, NeuroEndocrine Tumour)

A total of 364 participants were prospectively recruited between 2007 and 2017 across 9 North London hospitals. All the image acquisition was performed on the same machine at the University College London Hospitals (UCLH). Combined FDG-PET/CT perfusion imaging was successfully performed in 286 participants (M0colon: 123, M0rectum: 94 and M+: 69). The details of participant recruitment can be found in Fig. 2. The median follow-up time was 30.7 months (range: 0.5–116 months), during which time there were 84 deaths (M0colon: 20, M0rectum: 21 and M+: 43).

**Fig. 3:**
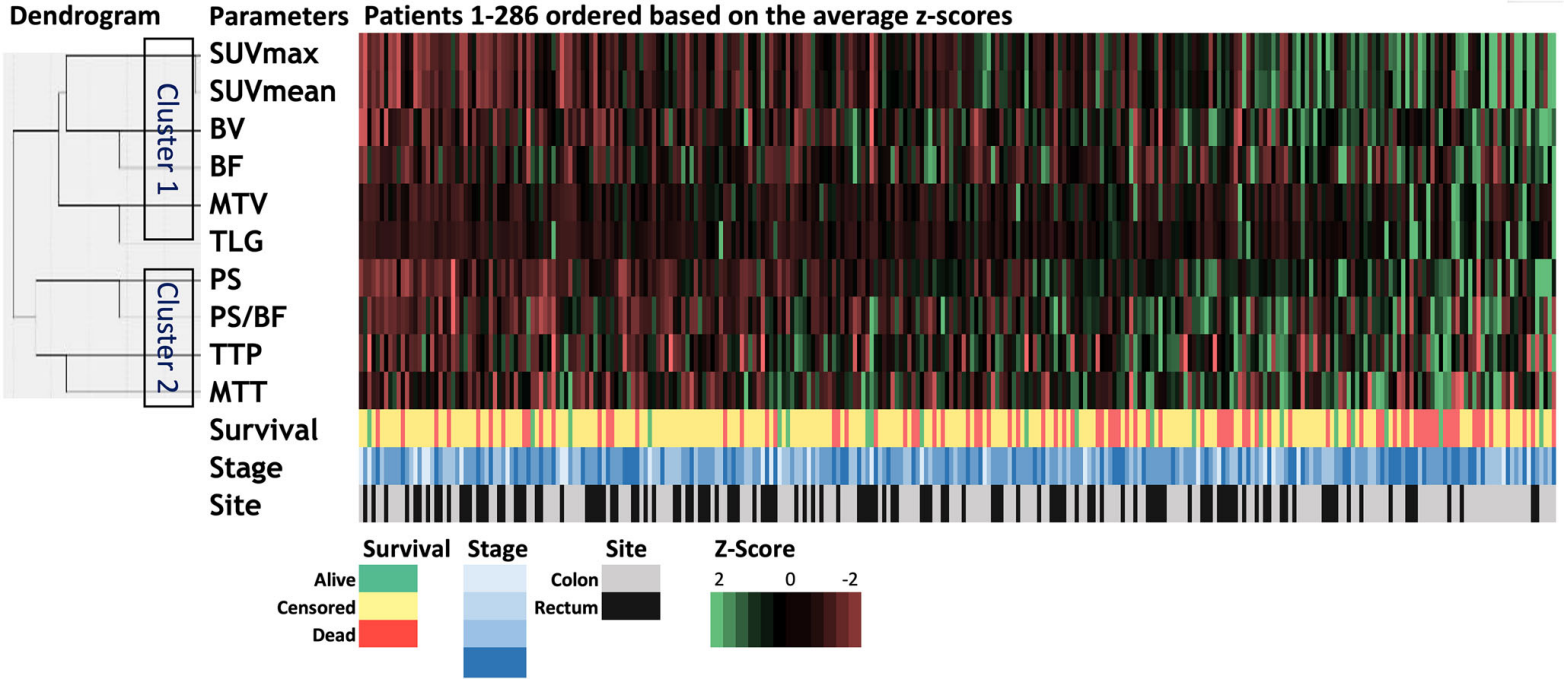
The cluster analyses displayed as a heatmap. The image parameters can be grouped into two clusters: one group consists of metabolic parameters as well blood flow and volume; the other is composed of vessel permeability and transit time measures. (SUV, Standardized Uptake Value; BV, Blood Volume; BF, Blood Flow; MTV, Metabolic Tumour Volume; TLG, Total Lesion Glycolysis; PS, Permeability Surface area product; PS/BF, ratio of PS over BF; TTP, Time To Peak; MTT, Mean Transit Time)

The heat map displaying the inter-relationships between vascular-metabolic features and clinical and outcome/survival is shown in Fig. 3. Cluster analysis revealed that the vascular and metabolic parameters could be grouped into two groups — Cluster 1 consists of SUV_max_, SUV_mean_, BV, BF, MTV, TLG; Cluster 2 is composed of PS, PS/BF, TTP, MTT (Fig. 3). The participants who survived less than 60 months appear to cluster towards the right which corresponds to a higher average z-score (i.e. higher vascular-metabolic features). No clear patterns were observed for tumour site or stage.

Table 1 displays the median values and ranges for vascular and metabolic parameters grouped by the two clusters within each clinical group. Significant differences were more often observed between M0colon and M0rectum as well as between M0rectum and M+.

**Table 1:** Lists the values of image parameters across the three clinical groups, as well as the statistical significance of the difference between the clinical groups for each image parameter

**Parameter**	**M0colon** (*n* = 123)	**M0rectum** (*n* = 94)	**M+** (*n* = 69)	***p*** **value**		
	Median (range)	Median (range)	Median (range)	M0colon vs M0rectum	M0colon vs M+	M0rectum vs M+
***Cluster 1***						
SUV_max_	16.4 (4.59–52.11)	14.4 (3.82–47.1)	15.4 (7.96–46.5)	.004	.875	.006
SUV_mean_	9.70 (2.63–32.1)	8.49 (2.50–19.5)	9.37 (4.18–28.3)	.002	.735	.012
BV (mL/100 mL)	8.29 (4.35–26.5)	7.77 (3.37–12.3)	7.84 (3.96–19.3)	<.001	.121	.091
BF (mL/min/100 mL)	67.9 (31.2–266.1)	63.4 (28.2–128)	67.3 (31.1–156)	.008	.928	.011
MTV (mL)	16.5 (1.95–246)	14.9 (1.24–85.5)	18.3 (4.63–280)	.222	.064	.012
TLG (L)	0.155 (0.010–2.262)	0.134 (0.004–0.725)	0.161 (0.028–2.479)	.020	.082	< .001
***Cluster 2***						
PS (mL/min/100 mL)	11.2 (1.78–40.7)	11.23 (5.29–47.7)	11.5 (5.68–33.5)	.781	.800	.999
PS/BF	0.15 (0.03–0.61)	0.18 (0.07–0.48)	0.16 (0.06–0.42)	.015	.859	.054
TTP (s)	40.6 (27.7–54.7)	42.7 (26.0–61.9)	42.2 (26.5–68.2)	.012	.061	.753
MTT (s)	11.2 (5.27–19.6)	11.3 (6.37–17.2)	11.1 (7.39–16.1)	.945	.086	.098

### Non-metastatic colon cancer

Table 2 lists the mean survival time for participants with non-metastatic colon cancers, after they were dichotomized according to the median value of the parameters. The median age of this group was 71 years. In non-metastatic colon cancer participants, age, tumour side and TLG were significantly associated with overall survival with Hazard Ratio (HR) of 1.046 (95% CI 0.995–1.099, log-rank test *p* = 0.044), 0.193 (95% CI 0.045–0.832, log-rank test *p* = 0.014) and 3.132 (95% CI 1.605–6.795, log-rank test *p* = 0.009) respectively.

**Table 2:** Results of univariate survival analysis for non-metastatic colon cancer participants. The participants were dichotomized according to the median value of each parameter. The mean and Standard Errors (SE) of the survival of the dichotomized groups were listed, as well as the Confidence Intervals (CI) of the hazard ratio

Parameter	Mean survival months ± SE > median (*N* = 61)	Mean survival months ± SE ≤ median (*N* = 62)	Log-rank *p* value	Hazard ratio	Lower 95% CI	Upper 95% CI
*Clinical*						
Age	85.44 ± 6.43	104.32 ± 5.01	0.044	1.046	0.995	1.099
Location	(right. *N* = 46) 110.56 ± 3.79	(left. *N* = 77) 84.03 ± 6.30	0.014	0.193	0.045	0.832
Stage	(I, II. N = 69) 103.25 ± 4.55	(III. *N* = 54) 75.42 ± 6.68	0.050	0.410	0.163	1.030
*Cluster 1*						
SUV_max_	92.48 ± 6.15	84.82 ± 4.15	0.133	1.032	0.984	1.083
SUV_mean_	94.73 ± 5.94	83.11 ± 4.33	0.375	1.054	0.974	1.141
BV	94.08 ± 6.42	80.38 ± 4.97	0.961	0.898	0.725	1.113
BF	93.26 ± 6.76	79.92 ± 4.82	0.911	0.992	0.974	1.010
MTV	87.75 ± 7.18	96.81 ± 6.64	0.132	1.011	1.004	1.018
TLG	87.31 ± 6.32	92.08 ± 5.07	0.009	3.302	1.605	6.795
*Cluster 2*						
PS	87.14 ± 7.73	96.16 ± 6.47	0.448	1.010	0.941	1.083
PS/BF	84.39 ± 7.11	101.96 ± 5.63	0.082	14.897	0.216	1026.387
TTP	90.73 ± 6.36	84.18 ± 4.54	0.680	0.987	0.918	1.061
MTT	94.92 ± 6.02	79.34 ± 5.46	0.490	1.014	0.845	1.217

**Fig. 4:**
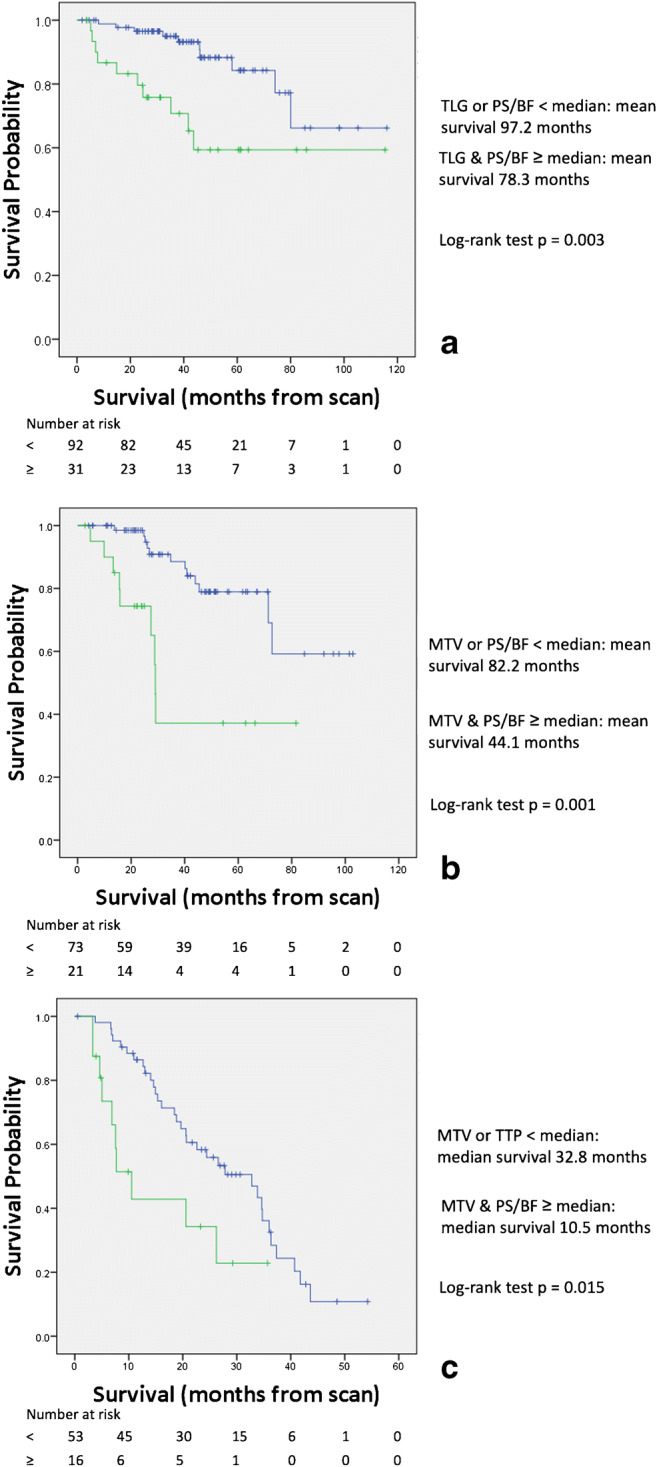
Kaplan–Meier curves for (**a**) M0col patients with both TLG and PS/BF above the median (lower, green line) versus others (upper, blue line), (**b**) M0rect patients with both MTV and PS/BF above the median (lower, green line) versus others (upper, blue line), and (**c**) M+ patients with both MTV & time-to-peak above the median (lower, green line) versus others (upper, blue line). (TLG, Total Lesion Glycolysis; MTV, Metabolic Tumour Volume; TTP, Time To Peak; PS, Permeability Surface area product; BF, Blood Flow)

Participant tumours that had both TLG & PS/BF above their respective median values had poorer survival than the others (*p* = 0.003, Fig. 4a), with mean survival 78.27 ± 9.37 months versus 97.16 ± 5.73 months. Following 5-fold cross-validation, the HR from Cox regression was 3.472 (95% CI: 1.441–8.333, log-rank test *p* = 0.006).

In a multivariate analysis consisting of age, tumour side and the combination of vascular-metabolic parameter (TLG combined with PS/BF), both tumour side and the combination of vascular-metabolic parameter were independent prognosticators (*p* = 0.033 and 0.018 respectively) (Table 3).

**Table 3:** Results of multivariate survival analysis for non-metastatic colon cancer participants taking the significant clinical parameter, tumour side and the vascular-metabolic parameters from univariate analysis

Parameter	*p* value	Hazard ratio	Lower 95% CI	Upper 95% CI
Age	0.184	1.034	0.984	1.087
Tumour side	0.033	0.203	0.047	0.879
Vascular-metabolic	0.018	2.92	1.203	7.142

**Fig. 5:**
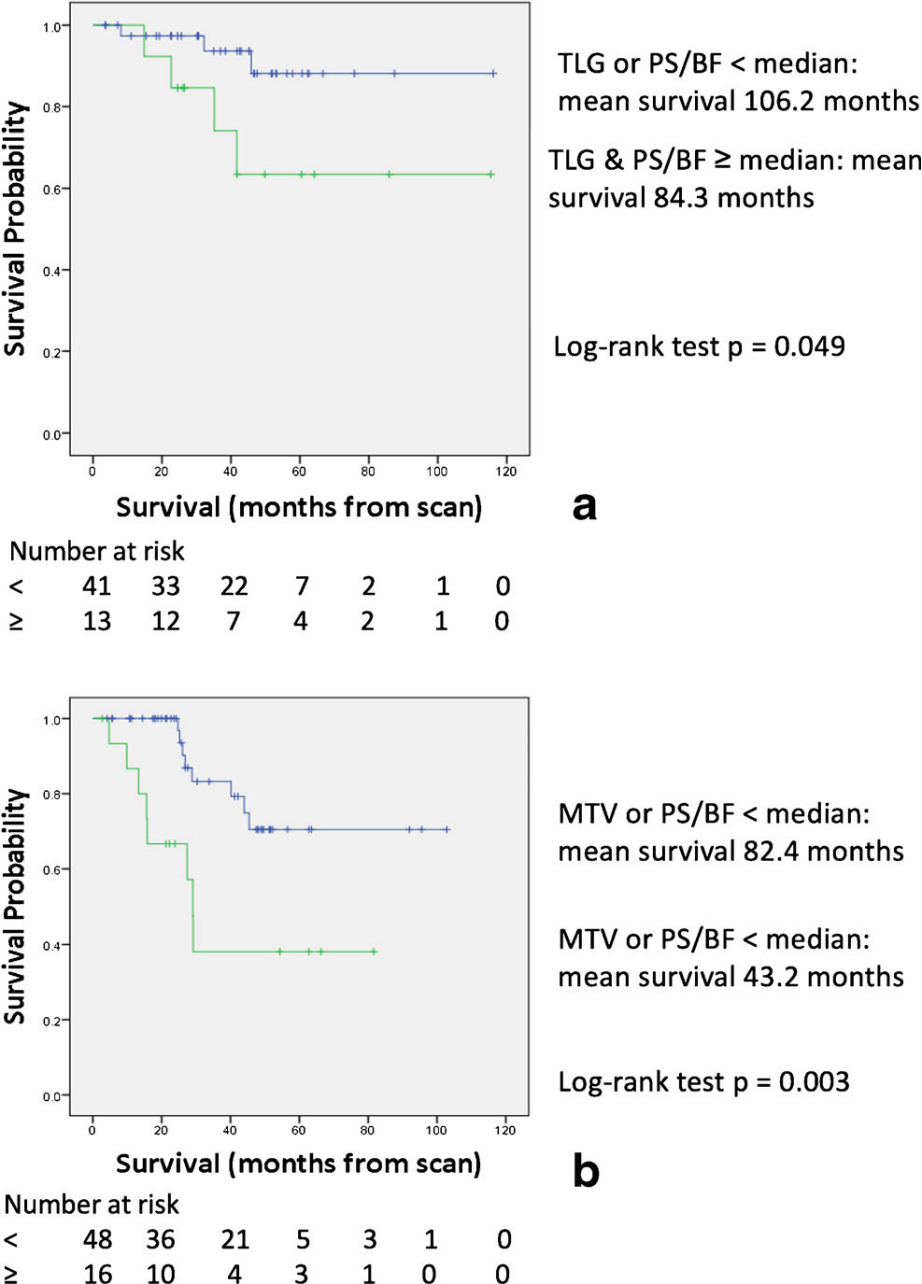
Kaplan–Meier curves for (**a**) colon cancer stage 2 patients with both TLG and PS/BF above the median (lower, green line) versus others (upper, blue line), (**b**) rectal cancer stage 3 patients with both MTV and PS/BF above the median (lower, green line) versus others (upper, blue line). The vascular-metabolic parameter could further stratify these patients into high and low risk groups. (TLG, Total Lesion Glycolysis; MTV, Metabolic Tumour Volume; PS, Permeability Surface area product, BF, Blood Flow)

The vascular-metabolic parameter (TLG combined with PS/BF) could further stratify stage 2 participants into good and bad prognosis (Fig. 5). Those with both TLG and PS/BF above the respective median values had worse survival than others (mean survival 84.25 ± 12.64 months versus 106.22 ± 5.43 months, log-rank test *p* = 0.049, Fig. 5a). In stage 3 participants, those with both TLG and PS/BF above the median values had mean survival of 58.44 ± 8.40 months, while the mean survival of others was 83.55 ± 7.00 (log-rank test *p* = 0.199). There were only 15 stage 1 participants and all of them survived during the follow-up period. Thus, no survival analysis was done on the stage 1 participants.

### Non-metastatic rectal carcinoma

Table 4 lists the mean survival time when the participants with non-metastatic rectal cancers, after they were dichotomized according to the median value of the parameters. The median age of this group was 69.5 years. In this group of participants, age and MTV were significantly associated with survival with HR of 1.081 (95% CI 1.028–1.137, log-rank test *p* = 0.035) and 1.050 (95% CI 1.025–1.076, log-rank test *p* = 0.024) respectively. The vascular parameter most closely associated with survival was PS/BF but not reaching statistical significance. Stratifying the participants by the median values of MTV and PS/BF, those with both MTV and PS/BF above their respective median values had poorer survival than the others (*p* = 0.001, Fig. 4b) with a mean survival of 44.05 ± 8.19 months versus 82.16 ± 5.24. Following 5-fold cross validation, the HR from Cox regression was 4.567 (95% CI: 1.901–10.970, log-rank test *p* = 0.001).

**Table 4:** Results of univariate survival analysis for non-metastatic rectal cancer participants. The participants were dichotomized according to the median value of each parameter. The mean and standard errors (SE) of the survival of the dichotomized groups were listed, as well as the confidence intervals (CI) of the hazard ratio

Parameter	Mean survival months ± SE > median (*N* = 47)	Mean survival months ± SE ≤ median (N = 47)	Log-rank *p* value	Hazard ratio	Lower 95% CI	Upper 95% CI
*Clinical*						
Age	67.16 ± 6.19	91.31 ± 5.33	0.035	1.081	1.028	1.137
Stage	(I, II. *N* = 30) 81.81 ± 7.04	(III. *N* = 64) 74.33 ± 5.78	0.129	0.466	0.170	1.277
*Cluster 1*						
SUV_max_	74.01 ± 6.37	81.67 ± 5.82	0.752	1.038	0.994	1.084
SUV_mean_	76.37 ± 6.23	79.37 ± 6.08	0.749	1.085	0.974	1.209
BV	77.53 ± 6.07	74.45 ± 6.30	0.497	0.799	0.619	1.032
BF	80.39 ± 7.45	71.78 ± 6.47	0.263	0.982	0.956	1.008
MTV	69.63 ± 7.31	82.39 ± 5.71	0.024	1.050	1.025	1.076
TLG	68.48 ± 7.53	82.19 ± 6.12	0.079	1.004	1.002	1.007
*Cluster 2*						
PS	75.06 ± 6.75	73.05 ± 7.05	0.803	1.029	0.934	1.135
PS/BF	69.13 ± 6.80	82.65 ± 7.07	0.084	46.702	0.376	5801.971
TTP	72.58 ± 6.48	72.13 ± 7.52	0.213	1.035	0.967	1.107
MTT	79.17 ± 5.63	52.92 ± 4.02	0.310	0.959	0.779	1.180

In multivariate analysis consisting of age, and the combination of vascular-metabolic parameter (MTV combined with PS/BF), both parameters were independent prognosticators (*p* = 0.002 and 0.001 respectively) (Table 5).

**Table 5:** Results of multivariate survival analysis for non-metastatic rectum cancer participants taking the significant clinical parameter and the vascular-metabolic parameters from univariate analysis

Parameter	*p* value	Hazard ratio	Lower 95% CI	Upper 95% CI
Age	0.002	1.087	1.030	1.148
Vascular-metabolic	0.001	4.630	1.901	11.236

The combination of MTV and PS/BF could further stratify stage 3 participants into either a good or poor prognosis (Fig. 5b). Those with both MTV and PS/BF above the respective median values had worse survival than the others (mean survival 43.19 ± 8.77 months versus 82.43 ± 6.14 months, log-rank test *p* = 0.003). The number of participants with stage 1 and 2 diseases were 10 and 20 respectively, too few for survival analysis.

### Metastatic colorectal carcinoma

Table 6 lists the mean survival time of the participants with metastatic colorectal cancers, after they were dichotomized according to the median value of the parameters. The median age of this group was 66 years. For metastatic colorectal cancer participants, the number of metastatic organs at presentation and TTP were significantly associated with survival on univariate analysis with HR of 2.572 (95% CI 1.320–5.011, log rank test *p* = 0.004) and 1.099 (95% CI 1.044–1.157, log rank test *p* = 0.030) respectively. The metabolic parameter most closely associated with survival was MTV, but did not reach statistical significance. Stratifying participants by the median values of MTV and TTP, those with high MTV and long time-to-peak, showed poorer survival than the others median survival of 10.52 ± 2.53 months versus 32.78 ± 5.85 months (*p* = 0.015, Fig. 4c). Following 5-fold cross-validation the HR from Cox regression was 2.421 (95% CI: 1.162–5.045, log rank test *p* = 0.018).

**Table 6:** Results of univariate survival analysis for metastatic colorectal cancer participants. The participants were dichotomized according to the median value of each parameter. The mean and standard errors (SE) of the survival of the dichotomized groups were listed, as well as the confidence intervals (CI) of the hazard ratio. The *Italics* font denotes metabolic parameters from the FDG-PET

Parameter	Mean survival months ± SE > median (*N* = 34)	Mean survival months ± SE ≤ median (*N* = 35)	Log-rank *p* value	Hazard ratio	Lower 95% CI	Upper 95% CI
*Clinical*						
Age	24.00 ± 2.56	29.36 ± 3.39	0.247	1.004	0.975	1.033
Location	(right. *N* = 14) 22.84 ± 3.68	(left. *N* = 55) 27.95 ± 2.59	0.280	1.446	0.737	2.839
Number of metastatic organs	(1–2. N = 54) 29.26 ± 2.57	(≧ 3. *N* = 15) 18.88 ± 2.86	0.004	2.572	1.320	5.011
*Cluster 1*						
*SUV*_*max*_	23.66 ± 2.79	28.73 ± 2.88	0.351	1.043	1.003	1.085
*SUV*_*mean*_	24.00 ± 2.78	28.35 ± 2.90	0.450	1.054	0.984	1.129
BV	30.16 ± 3.03	22.57 ± 2.51	0.097	0.929	0.784	1.100
BF	23.62 ± 2.34	27.92 ± 2.97	0.306	1.005	0.991	1.019
*MTV*	23.39 ± 2.84	28.70 ± 2.69	0.239	1.005	0.999	1.012
*TLG*	24.37 ± 2.75	27.88 ± 2.79	0.462	1.001	1.000	1.001
*Cluster 2*						
PS	25.57 ± 2.68	26.46 ± 2.83	0.865	0.958	0.890	1.030
PS/BF	25.69 ± 2.69	26.77 ± 2.89	0.917	0.161	0.003	9.529
TTP	21.21 ± 2.33	30.27 ± 2.93	0.030	1.099	1.044	1.157
MTT	25.93 ± 2.82	25.60 ± 2.49	0.786	0.943	0.798	1.115

In multivariate analysis consisting of the number of involved organs, and the combination of vascular-metabolic parameter (MTV combined with TTP), both parameters were independent prognosticators (*p* = 0.012 and 0.021 respectively) (Table 7).

**Table 7:** Results of multivariate survival analysis for metastatic colorectal cancer participants taking the significant clinical parameter and the vascular metabolic parameters from univariate analysis

Parameter	*p* value	Hazard ratio	Lower 95% CI	Upper 95% CI
Number of metastatic organs	0.012	2.347	1.204	4.577
Vascular-metabolic	0.021	1.459	1.058	2.013

### Inter-observer agreement

The inter-observer agreement for image parameters is shown in Table 8. There was moderate to good agreement with ICC values greater than 0.7. The COV was below 33% for vascular and metabolic parameters.

**Table 8:** Intra-class Correlation Coefficient (ICC), its confidence interval and Coefficient of Variation (CV) of image parameters

Parameter	ICC	95% confidence interval	CV (%)
*Cluster 1*			
SUV_max_	0.997	0.993–0.999	2.94
SUV_mean_	0.833	0.668–0.920	20.89
BV	0.794	0.647–0.885	27.43
BF	0.933	0.860–0.969	15.35
MTV	0.842	0.612–0.934	32.26
TLG	0.968	0.939–0.983	25.78
*Cluster 2*			
PS	0.893	0.784–0.948	18.77
PS/BF	0.863	0.765–0.922	22.31
TTP	0.700	0.453–0.848	15.87
MTT	0.740	0.497–0.875	14.67

## Discussion

This study reports metabolic and vascular data from a large cohort of colorectal carcinoma participants, many of which have had extensive follow-up. The significant differences in tumour vascular and metabolic parameters demonstrated for disease location (colon versus rectum) and tumour stage (M0 versus M+) confirm that imaging-derived tumour biology varies with these clinical scenarios, thereby supporting the need for prognostic markers that are specific to each situation. This also reflects the biological differences between colon and rectal cancers, such as different gene expression profiles including those involved in the metabolic pathways [5]. This study has also affirmed the potential for vascular-metabolic imaging with FDG-PET/CT to provide these site- and stage-specific markers of prognosis for colorectal participants. It was found that different combinations of vascular and metabolic parameters were required to optimize prognostic performance for each of the three clinical groups. This could help clinical judgement, if the merit of the particular vascular-metabolic profile is confirmed in further larger trials.

In this cohort, serial participants referred for staging before commencement of cancer treatment were recruited. The imaging studies were executed on the same scanner at UCLH and followed the exact protocol over the study period, minimizing the biases which might arise from using different machines and reconstruction parameters. As this was an observational study, the treatment of each participant was decided by the local team, reflecting their local preferences and respective guidelines independent of the vascular-metabolic image analysis undertaken in this study. This study data therefore provided a solid platform, to investigate the relationship of robust image parameters to the clinical outcomes in routine daily practice.

The authors have been able to identify only two prior studies reporting vascular-metabolic relationships in primary colorectal cancer (as opposed to metastatic lesions). Neither of these publications related imaging findings to survival. One study reported SUV_max_, BF, BV and PS values in 32 participants with rectal cancer. No association between image parameters and subsequent response to chemoradiation was found in this study [27]. Another study reported SUV_max_ and BF in a series of 45 participants with colorectal cancer [28]. In this series, there was association between vascular-metabolic phenotype and Vascular Endothelial Growth Factor (VEGF) expression, a known histological marker for poorer prognosis for participants with colorectal cancer [29]. Differences in vascular-metabolic relationships between early stage and advanced tumours were also observed [28]. This is a finding that adds further support for the need for imaging markers of prognosis to be stage-specific. The values this study recorded for these parameters are consistent with the former two studies. The favourable inter-observer agreement described for CT perfusion parameters is also comparable to other published data [30].

In this study, the image parameters derived from the FDG-PET and perfusion CT were divided into two clusters. One cluster consists of metabolic parameters from the FDG-PET together with blood flow and blood volume from the CT perfusion. The results from this study are compatible with the conclusions drawn by Goh et al., who found that the flow and metabolism were related in advanced colorectal cancer [28]. The other cluster is composed of permeability (PS, PS/BF) and transit time measures (TTP, MTT). This finding reflects the fact that tumours in which greater amounts of contrast material pass into the extravascular space will enhance for a longer period of time [19]. Combination of the metabolic and vascular parameters from these two clusters was able to identify a subset of participants who had worse survival in comparison to other participants in each subgroup.

In non-metastatic colorectal cancer, the vascular parameter found to be of prognostic significance when combined with a measure of tumour metabolism was PS/BF, which reflects not only blood flow but also vascular permeability. When a tumour vascular supply exhibits a flow-limited regime such that BF greatly exceeds PS, PS/BF will approximate the extraction efficiency of contrast material [19]. The tumour BF significantly exceeded PS in all of the cases in this study (median values for PS/BF 0.15–0.18, Table 2) indicating that a flow-limited regime is typically found in colorectal cancer. Extraction efficiency (as represented by PS/BF) represents the leakiness of tumour blood vessels independent of blood flow, which in turn is likely to reflect the degradation of the capillary basement membrane induced by VEGF expression.

In participants with distant metastases, the number of involved metastatic organs was prognostic. The more organs involved, the worse the survival. In addition, the vascular parameter of prognostic significance alone and in combination with a measure of metabolism was TTP. A previous study of 53 participants with colorectal cancer found this parameter to be positively correlated with tumour stage with high-grade tumour demonstrating higher TTP [31]. Another report also found higher TTP values in poorly-differentiated tumours in a series of 37 CRC participants [32]. TTP is determined by several physiological factors related to the tumour vascular support, including BV, PS and extracellular fluid volume. The multivariate analysis revealed that the vascular-metabolic profile and the number of metastatic organs were independent prognosticators.

The two metabolic parameters found to be of prognostic significance in this research study were MTV and TLG, both of which are already recognized as markers of tumour aggressiveness. In a series of 138 colorectal participants undergoing surgical resection, tumours with higher MTV tended to metastasize, and the predictive value of MTV was better than SUV_max_ [33]. Their roles as prognosticators were also demonstrated in another report consisting of 64 rectal cancer participants treated with concurrent chemoradiotherapy, MTV of the baseline PET as well as changes of TLG were prominent prognosticator in multivariate Cox regression analysis [34].

The possible clinical applications of prognostic imaging biomarkers in colorectal cancer will be specific to each clinical group considered in this research study. For participants with non-metastatic colon cancer, prognostic markers could feasibly identify a high-risk sub-group of participants with stage 2 disease who might benefit most from the adjuvant chemotherapy. Current clinical guidelines consider a range of pathological features that may be taken into account when identifying high-risk patients [35]. Vascular-metabolic markers could feasibly provide an additional risk factor to be used in conjunction with these pathological markers, prompting adjuvant chemotherapy in otherwise low risk patients, or escalation of treatment in those considered to be high-risk. Adjuvant chemotherapy is also recommended for all patients with stage 3 disease and typically comprises of oxaliplatin-based regimens. Oxaliplatin exposure carries the risk of sensory neuropathy. Prognostic markers may be able to identify low-risk participants, for whom de-escalation of treatment by shortening the length of their chemotherapy treatment, could reduce the exposure to oxaliplatin and resultant risk of sensory neuropathy [36].

Following completion of initial treatment, participants with non-metastatic colorectal typically enter a surveillance regime to facilitate early detection of tumour recurrence. Risk-adapted surveillance in which the intensity of follow-up is determined by prognostic markers can improve the targeting of curative re-operations and reduce the costs of surveillance for disease-free patients [37].

The optimal management of metastatic colorectal cancer is evolving as data accumulates. With advances in chemotherapy and surgery, there is an incentive to pursue aggressive management in selected patients to achieve long-term survival. Resection of liver or pulmonary metastatic disease is accepted practice and can produce survival benefit [38, 39]. However, the role of surgery is less defined in patients with more extensive metastases, such as concurrent liver and lung metastases [40, 41]. With advances in modern chemotherapeutic options, there is current debate as to whether surgical or medical approach would be best for any given patient. Prognostic imaging markers could potentially contribute to the personalization of treatment for these patients.

Limitations of this study include the fact that imaging was performed in a single tertiary institution. Multi-centre studies are required to confirm these findings and to demonstrate the practicality of performing FDG-PET/CT perfusion imaging in non-specialist institutions. The participants that comprised in this study were from 9 different hospitals (but scanned at one hospital). The researchers only had sight of their overall survival (courtesy of their long follow-up at the referring hospital or general practice) which was the main outcome of this prospective study protocol. The authors accept that the clinical practice in each referring hospital differed and this study did not have access to detailed follow-up information (required to assess accurate time to progression or response to treatment). Consequently, this study was unable to evaluate these additional important outcome measures. This extra data may be able to provide additional insights into the utility of the vascular-metabolic parameter in specific context of different targeted therapies employed in clinical practice and management of colorectal cancer patients. This could perhaps be further evaluated in future studies. Nearly 15% of participants were excluded from the analysis due to failure of the FDG-PET/CT perfusion study. It was recognised that CT perfusion for colorectal cancer is constrained by the motion of respiration and bowel [42]. In this cohort, this limitation affected not only tumours closest to the diaphragm (transverse colon) but also tumours at ascending, descending, sigmoid colon and rectum. The failure rate in this cohort was similar to that in the literature and reflected this technical challenge. It potentially could be improved by adopting motion correction in the future versions of the processing software, or using breath-hold monitoring with patient feedback [43]. Furthermore, the failure rate is comparable with the concordance rate of KRAS mutation tests using frozen samples being only 83% [44]. As surgical specimens were not consistently available in this cohort, this study has been unable to seek further relationship between vascular-metabolic profiles and histopathologic features. It was also unable to compare the prognostic performance of vascular-metabolic profiles to well-known pathological risk factors such as tumour regression score, circumferential resection margin, lymphovascular/perineural invasion, microsatellite instability, KRAS, NRAS, BRAF mutations. These limitations will need to be addressed in future studies.

In summary, this study demonstrates the potential for vascular-metabolic imaging with FDG-PET/CT perfusion to provide markers of prognosis specific to colorectal cancer location and stage. Combined vascular-metabolic parameters afford better markers of prognosis than individual vascular or metabolic parameters deployed alone. The findings are potentially clinically relevant enabling a tailored precision medicine approach to colorectal cancer.
